# Treatment decision‐making for a post‐traumatic malocclusion in an elderly patient: A case report.

**DOI:** 10.1111/edt.12673

**Published:** 2021-03-18

**Authors:** Jude Ugochukwu Amadi, Filippo Delitala, Gianmauro Liberatore, Emanuele Scozzafava, Bruno Carlo Brevi

**Affiliations:** ^1^ Maxillofacial Surgery Unit Azienda Ospedaliero Universitaria Pisana Pisa Italy; ^2^ Department of Maxillofacial Surgery University of Siena Siena Italy

**Keywords:** bilateral sagittal split osteotomy, condylar fracture, geriatric trauma, post‐traumatic malocclusion

## Abstract

Traumatic dental injuries in elderly patients are a rising trend due to demographic and social changes of the population. Older dentulous patients in good health have become increasingly common. The development of a post‐traumatic malocclusion is a common sequela resulting from mandibular condyle fracture, as in the case reported in this paper. The decision‐making process led the authors to rule out conservative treatment options and to perform orthognathic surgery on an 81‐year‐old patient, an unprecedented report in the literature. At one‐year follow‐up, prophylactic therapy, a specific surgical technique, and osteotomy fixation have restored the occlusion to the pre‐traumatic condition.

## 1 INTRODUCTION

Over the past 40 years, the incidence of maxillofacial trauma in elderly patients has been constantly increasing. The causes can be a lengthening in life expectancy and a more active lifestyle, which both expose the elderly to a higher risk of trauma.^1^

Mandibular condyle fractures, mostly due to falls and motor vehicle accidents, are common among the elderly and often result in malocclusion.^2^, ^3^ Different treatment strategies can be adopted for this condition, depending on the characteristics of both the fracture and the patient.

This report concerns a case of an 81‐year‐old male patient with an unsuccessfully treated bilateral condylar fracture who underwent surgical intervention in order to correct his post‐traumatic malocclusion.

## 2 CASE REPORT

The PRICE guidelines were followed in this case report. Written informed consent was obtained from the patient.

An 81‐year‐old male was admitted to the authors’ department outpatient clinic with a history of facial trauma caused by an accidental fall that occurred 10 months before. Immediately after the trauma, the patient was admitted to another hospital emergency department where clinical examination and a CT scan confirmed bilateral condyle fractures. No surgical treatment was provided. Soft diet, non‐specific rehabilitation therapy and management with NSAIDs and a muscle relaxant were prescribed. Six weeks later, during a subsequent examination, a post‐traumatic malocclusion was noted. Mandibular exercises and a dental examination were prescribed.

**FIGURE 1:**
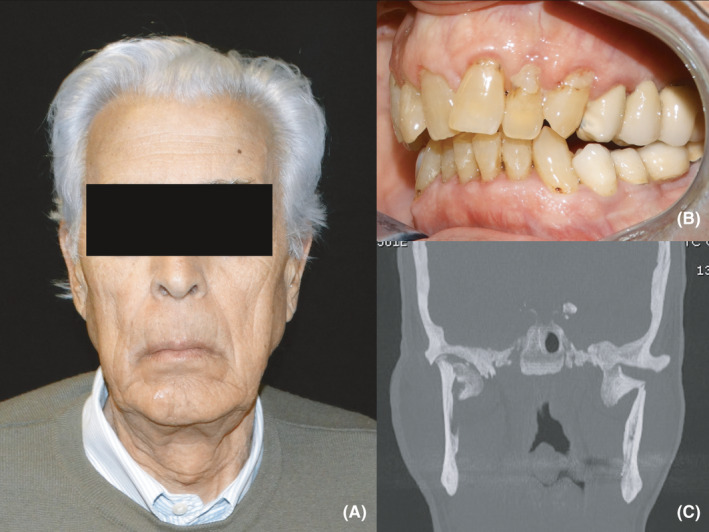
The patient at the first clinical examination. (A) Frontal view. (B) Intraoral photograph shows dentulous upper and lower arches, increased overjet and mild anterior open bite. (C) Coronal CT scan demonstrates bilateral condylar fractures

The physical examination, conducted 10 months after the trauma, showed dentulous upper and lower arches, post‐traumatic malocclusion with an increased overjet, and a slight anterior open bite without restrictions in the mandibular range of motion (Figure 1). The patient reported mild discomfort during mastication but no pain.

Given his characteristics of being elderly and almost fully dentulous, the patient was initially referred to his dentist for occlusal equilibration therapy. However, given its complexity due to the significant loss of mandible height, which would have implied extensive molar grinding, the dentist advised the patient against such treatment. In addition, occlusal equilibration would have not managed the mandibular retrusion and would not have restored the pre‐trauma occlusion.

Since the patient strongly demanded treatment to address the retruded appearance of his mandible, surgical correction of the malocclusion with orthognathic surgery involving the lower jaw was suggested. An orthopantogram and a lateral cephalogram of the patient were obtained. Standard model planning was done, and supplemental calcium and vitamin D therapy were prescribed, starting from ten days prior to the surgical intervention.

**FIGURE 2:**
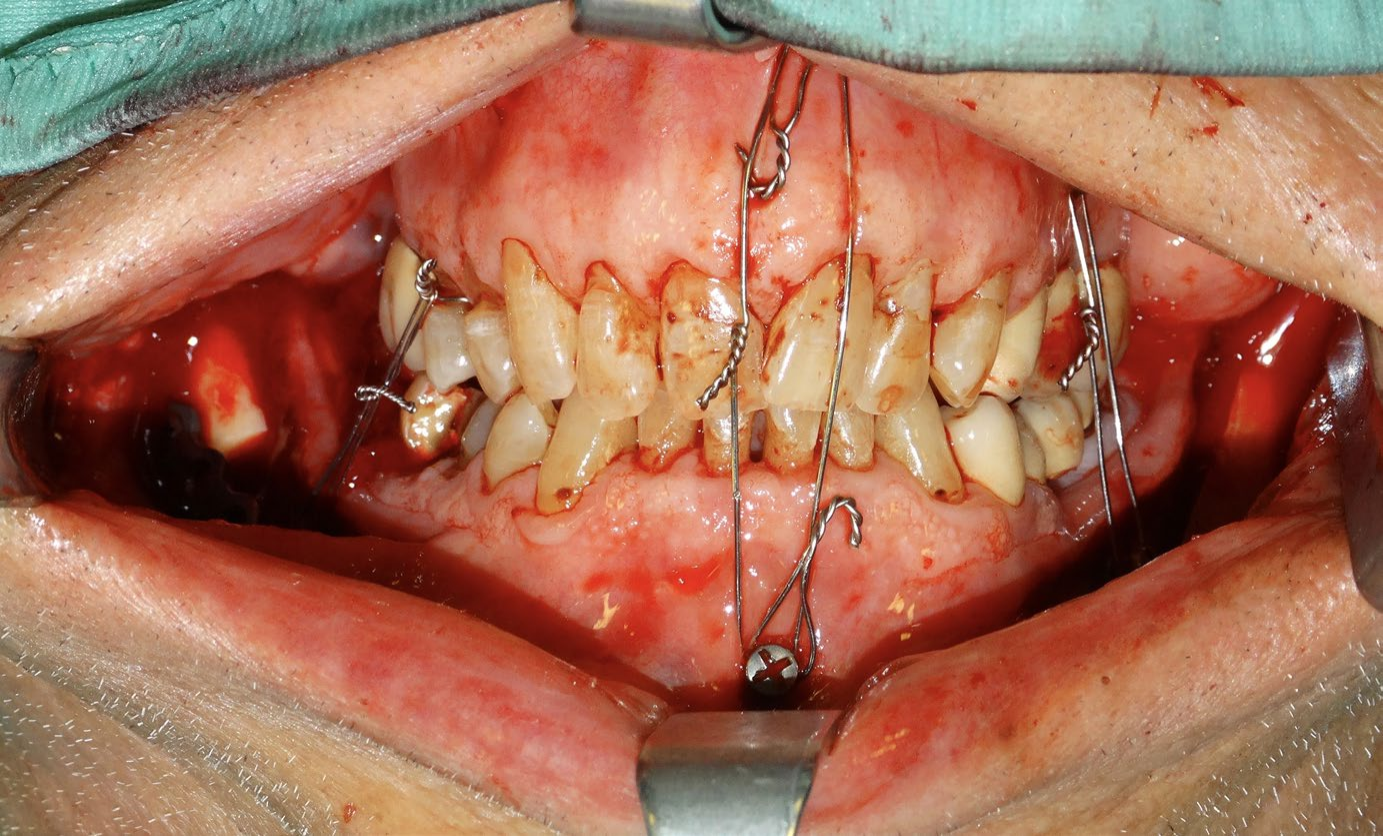
Intra‐operative view showing the intermaxillary fixation. Spino‐mental screws were used for intra‐operative IMF, and wire hooks were used for potential postoperative elastic IMF

A bilateral sagittal split osteotomy (BSSO) was performed to advance and rotate the mandible in order to restore the occlusion. Intermaxillary fixation (IMF) was accomplished with both tooth‐ and bone‐borne appliances. Spino‐mental fixation was applied with two trans‐mucosally inserted self‐drilling screws. Two S‐shaped wire hooks were attached to the central IMF screws (Figure 2), and two IMF screws were applied on the left side. On the right side, fixation was achieved with an IMF screw in the mandibular bone and with a wire ligature on a maxillary premolar. A single 2.3 plate was applied to each mandibular side for internal fixation.

**FIGURE 3:**
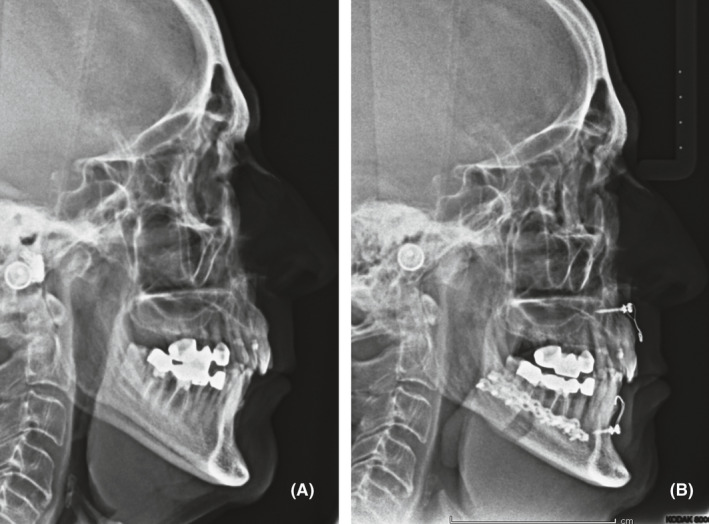
Preoperative (A) and 48 h postoperative (B) lateral cephalograms showing the advancement and counterclockwise rotation of the mandible

**FIGURE 4:**
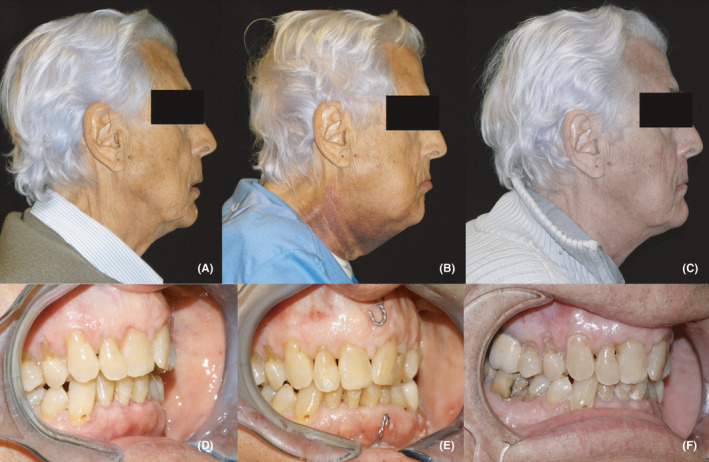
Lateral and intraoral photographs. (A, D) Preoperative. (B, E) At 48 h after surgery showing correction of the malocclusion and extensive cervical bruising. (C, F) At one‐year follow‐up, the occlusion remained stable

Spino‐mental hooks were left in place after the surgery for potential elastic fixation, in order to provide occlusal guidance and to lighten the condylar load consequent to mandibular advancement and rotation, thus avoiding condylar resorption (Figure 3). Besides persistent cervical bruising, there were no major post‐operative complications and the patient was discharged from the hospital three days after the surgery. A non‐steroidal anti‐inflammatory drug was prescribed, along with continuing the supplemental calcium and vitamin D therapy for 2 months. Follow‐up visits were scheduled up to 1 year after intervention. The spino‐mental screws were removed after two weeks. No further dental or prosthetic treatments were needed, and the occlusion remained stable over time (Figure 4).

## 3 DISCUSSION

A lengthening in life expectancy has increased the percentage of the elderly within the general population, and a life quality improvement with a transition to a more active lifestyle has led to a rise in the incidence of trauma in the geriatric population.^1^ Management of trauma in elderly patients has become a rising trend because it represents almost 23% of all traumatic injuries.^4^ When compared to a younger population, the combination of comorbidities, limited physiological reserves, and overall frailty make older patients more vulnerable to traumatic injuries and to their complications.^5^ Mandibular fractures, especially in the condylar process, are frequent and often caused by accidental falls combined with the intrinsic characteristics of bone structure in the elderly.^2^, ^3^ Condylar fracture treatment has raised many debates in the literature.^6^, ^7^, ^8^ The principles of treatment for the geriatric population are the same, although influenced by specific factors such as bone atrophy, reduced capacity for tissue repair and concurrent diseases. Anatomical hazards of open treatment, satisfactory results of closed treatments, and the functional rather than esthetic goals make non‐surgical treatment more frequent among geriatric patients,^9^ as recently reported by a European multicenter study.^10^ Regardless of whether the initial choice is an open or closed treatment, condylar fractures are burdened with a significant rate of unsuccessful outcomes as in the presented case. Successful treatment in condylar fractures depends on the biological characteristics and adaptive capability of the patient's masticatory system, which differs between elderly and younger individuals. The elderly's lack of sound biology and adaptation can lead to an unfavorable outcome even with good treatment, especially in cases with bilateral fractures which require a more extensive adaptation.^11^

**FIGURE 5:**
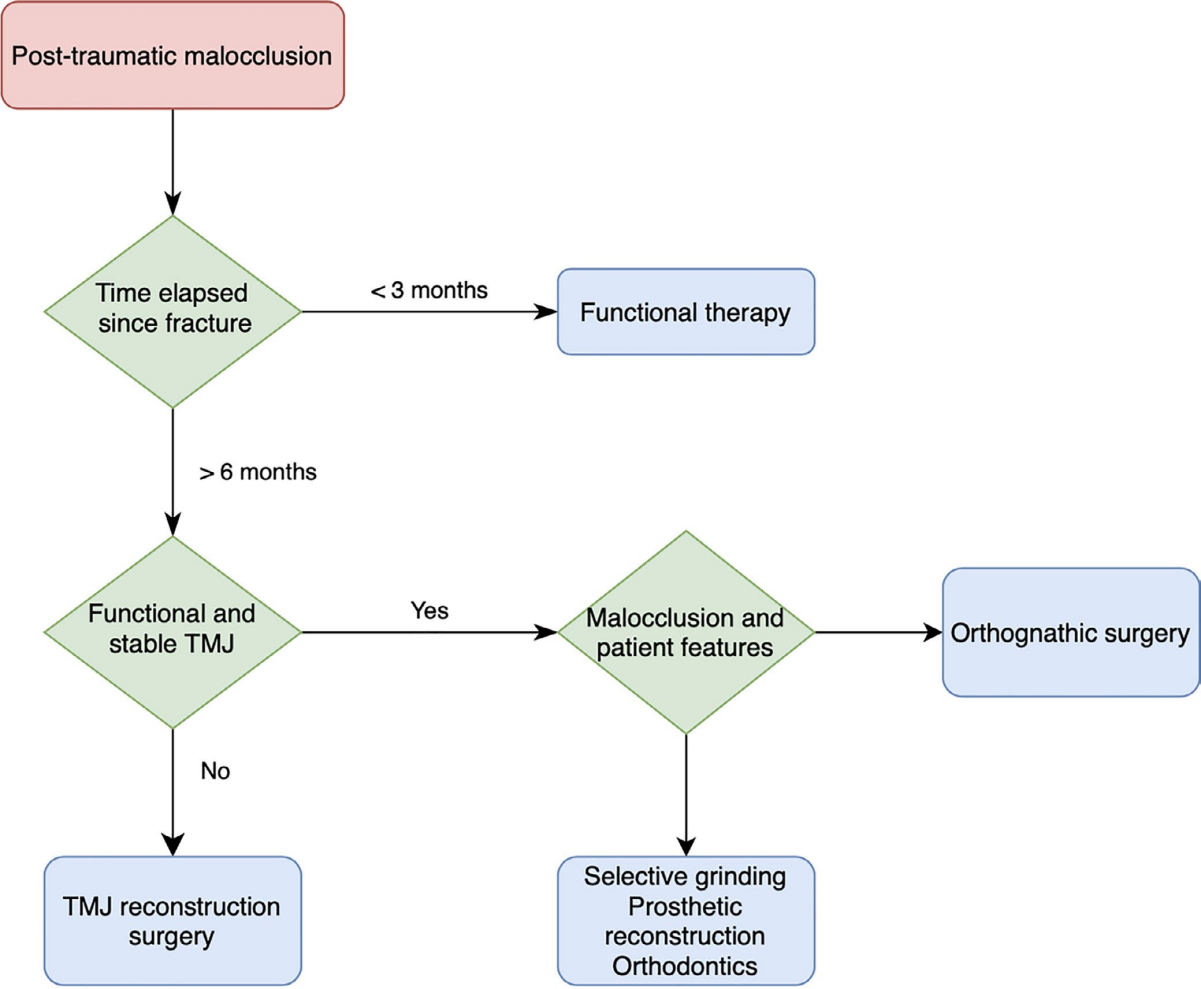
Treatment strategy for malocclusions resulting from condylar fractures

Malocclusion is one of the most common complications after treating patients with condylar fractures, with an incidence ranging from 1.4% to 13.5% of cases.^12^, ^13^ One of the most significative factors for such a complication is the degree of mandibular ramus deformity. Many treatment strategies are available depending on the severity, the location and age of the fracture, and on the patient characteristics. The goals of therapy, regardless of the type of treatment, are the achievement of a stable occlusion, and regular mandibular function and shape. Mandibular ramus and temporomandibular joint (TMJ) conditions are the most important variables to consider when choosing the secondary treatment: Cases with severe ramus shortening or fragmentation, or with poor TMJ function due to ankylotic, necrotic, or resorptive processes, are best managed with TMJ reconstruction surgery, although it is a less frequent scenario.^14^ Once good TMJ function is established, post‐traumatic malocclusion can be addressed with both conservative and surgical treatments. Time elapsed between the trauma and its late correction is a significant factor in choosing the treatment. Functional rehabilitation can be an effective therapy in the first months after the initial injury, while TMJ, bony and soft tissue remodeling processes are still occurring.^15^ After about three months, it is uncommon to achieve a successful outcome using functional therapy. In such cases, it is crucial to take into account the type of fracture, and the patient's concerns and biological characteristics. Mild post‐traumatic malocclusions in patients with good dental and periodontal health can be treated with occlusal equilibration, prosthetic reconstruction, or orthodontics. These conservative treatments, based on changes in teeth shape and position, can correct post‐traumatic malocclusions without posing particular risks. In patients with a good performance status, repositioning the maxillo‐mandibular complex with orthognathic surgery can correct more severe malocclusions, such as post‐bilateral condylar fracture anterior open bite (Figure 5).

Given the current case characteristics, of an 81‐year‐old dentate patient with a 10‐month‐old bilateral condylar fracture, good mandibular function with no pain and indications of a stable craniomandibular articulation, who presented with an anterior open bite malocclusion with increased overjet, the authors first suggested a conservative approach by means of occlusal equilibration. Two studies about the correction of anterior open bite with occlusal equilibration have reported mixed outcomes, showing 33.3% relapse and a risk of dentine hypersensitivity related to the extent of occlusal grinding.^16^, ^17^ These findings indicate that occlusal equilibration can be more appropriate in the correction of minor malocclusion. This patient would have needed extensive grinding in order to balance the loss of ramus height. In addition, occlusal equilibration would not have resolved the loss of projection of the mandible which was one of his main complaints, nor it would have restored the pre‐trauma occlusion. Other conservative treatments, such as functional rehabilitation and orthodontics, were ruled out. Age and the bilateral fractures precluded the patient from the possibility of correcting the malocclusion with functional rehabilitation, while the presence of prosthetic appliances and a poor periodontal condition excluded orthodontic correction.

Several studies have reported the successful and predictable outcomes that orthognathic surgery produces in patients with long‐standing post‐traumatic malocclusion.^14^, ^18^, ^19^ The time interval between the injury and malocclusion is a paramount element in treatment decision: Malocclusions present for less than 3 months after injury can be treated as a fresh fracture, while those present for more than 6 months, and with a good mandibular range of motion and stable craniomandibular articulation, can be approached as a standard orthognathic surgery case.^14^ The site of the fractures and the resultant deformities dictate the type of osteotomy to use. In many studies, malocclusion resulting from bilateral condylar fractures is treated with either a bilateral sagittal split osteotomy of the mandible (BSSO), a Le Fort I osteotomy of the maxilla or a bimaxillary osteotomy.^14^, ^18^, ^19^ From a safety standpoint, a maxillary osteotomy poses a lower surgical risk. Nevertheless, posterior maxillary repositioning is not a simple procedure to perform. It might cause narrowing of the upper airway with concomitant airway volume decrease,^20^ and it is not capable of restoring the pre‐traumatic skeletal profile. The standard therapeutic decision in similar cases for younger patients is certainly a BSSO with mandibular advancement and counterclockwise rotation.

The authors’ main concern about performing a BSSO in this case was the patient's age. To the best of the authors’ knowledge, there are no studies in the literature describing orthognathic procedures on an 81‐year‐old patient. The misgivings were related to both technical and biological matters: the cancellous bone response to the sagittal split and its likelihood to follow Obwegeser's classic osteotomic line, the extent of mandibular rami vascularization after the osteotomy and the probability of resultant bone avascular necrosis, altered consolidation or recurrent infections.

In the literature, the few studies that focus on the outcome of orthognathic procedures on elderly patients yield mixed reports. August et al.,^21^ Ylikontiola et al.,^22^ and Parton et al.^23^ reported an age‐related increased incidence of postoperative neurosensory disturbance. Peacock et al. found an average longer hospitalization time and increased likelihood of hardware removal in patients older than 40 years compared to a younger group.^24^ Kriwalsky et al. showed that older patients were more at risk of a bad split than the younger ones.^25^ Conversely, Sloane et al. found no significative differences in the outcome between a group of patients older than 35 and a group of younger patients.^26^ Avelar et al. showed good post‐operative quality of life questionnaire scores in patients older than 60 years who underwent orthognathic procedures.^27^

A technical expedient that sets this case apart from younger patients’ cases is the use of a single 2.3 plate per side. In the authors’ center experience, when further mandibular fixation stability is required, two 2.0 plates per side are used. In this case, however, as mentioned earlier, concerns arose about the vascularization of the mandible after the split, especially in the distal area of the proximal segment. The use of one thicker plate per side increased stability and avoided excessive drill perforations on the cortical bone of both osteotomy sides, thus reducing the risk of complications related to poor local perfusion. Supplementary therapy with calcium and vitamin D was continued for 2 months after the surgery because of their beneficial effects contrasting physiological decrease of vitamin D synthesis due to increasing age.^28^ The patient's post‐operative period did not differ significantly from the usual post‐operative period of younger patients.

## 4 CONCLUSIONS

The present case discusses the therapeutic alternatives in an 81‐year‐old patient with a post‐traumatic malocclusion with preserved TMJ function.

## 5 AUTHOR CONTRIBUTION

**Jude Ugochukwu Amadi**: Conceptualization (lead); Visualization (lead); writing ‐ original draft (lead); writing ‐ review and editing (equal). **Filippo Delitala**: review and editing (equal). **Gianmauro Liberatore**: review and editing (equal). **Emanuele Scozzafava**: review and editing (equal). **Bruno Carlo Brevi**: Conceptualization (supporting); Supervision (lead); review and editing (equal).

## CONFLICT OF INTEREST

None.

## Data Availability

Data sharing is not applicable to this article as no new data were created or analyzed in this study.
